# Multi-omics reveals novel prognostic implication of SRC protein expression in bladder cancer and its correlation with immunotherapy response

**DOI:** 10.1080/07853890.2021.1908588

**Published:** 2021-04-08

**Authors:** Wenhao Xu, Aihetaimujiang Anwaier, Chunguang Ma, Wangrui Liu, Xi Tian, Maierdan Palihati, Xiaoxin Hu, Yuanyuan Qu, Hailiang Zhang, Dingwei Ye

**Affiliations:** a Department of Urology, Fudan University Shanghai Cancer Center, Shanghai, PR China; b Department of Oncology, Shanghai Medical College, Fudan University, Shanghai, PR China; c Department of Neurosurgery, Fudan University Shanghai Cancer Center, Shanghai, PR China; d Department of Radiology, Fudan University Shanghai Cancer Center, Shanghai, PR China

**Keywords:** Bladder cancer, biomarker, prediction model, multi-omics, SRC, immune checkpoint therapy

## Abstract

**Purpose:**

This study aims to identify potential prognostic biomarkers of bladder cancer (BCa) based on large-scale multi-omics data and investigate the role of SRC in improving predictive outcomes for BCa patients and those receiving immune checkpoint therapies (ICTs).

**Methods:**

Large-scale multi-comic data were enrolled from the Cancer Proteome Atlas, the Cancer Genome Atlas and gene expression omnibus based on machining-learning methods. Immune infiltration, survival and other statistical analyses were implemented using R software in cancers (*n* = 12,452). The predictive value of SRC was performed in 81 BCa patients receiving ICT from aa validation cohort (*n* = 81).

**Results:**

Landscape of novel candidate prognostic protein signatures of BCa patients was identified. Differential BECLIN, EGFR, PKCALPHA, ANNEXIN1, AXL and SRC expression significantly correlated with the outcomes for BCa patients from multiply cohorts (*n* = 906). Notably, risk score of the integrated prognosis-related proteins (IPRPs) model exhibited high diagnostic accuracy and consistent predictive ability (AUC = 0.714). Besides, we tested the clinical relevance of baseline SRC protein and mRNA expression in two independent confirmatory cohorts (*n* = 566) and the prognostic value in pan-cancers. Then, we found that elevated SRC expression contributed to immunosuppressive microenvironment mediated by immune checkpoint molecules of BCa and other cancers. Next, we validated SRC expression as a potential biomarker in predicting response to ICT in 81 BCa patient from FUSCC cohort, and found that expression of SRC in the baseline tumour tissues correlated with improved survival benefits, but predicts worse ICT response.

**Conclusion:**

This study first performed the large-scale multi-omics analysis, distinguished the IPRPs (*BECLIN, EGFR, PKCALPHA, SRC, ANNEXIN1* and *AXL*) and revealed novel prediction model, outperforming the currently traditional prognostic indicators for anticipating BCa progression and better clinical strategies. Additionally, this study provided insight into the importance of biomarker SRC for better prognosis, which may inversely improve predictive outcomes for patients receiving ICT and enable patient selection for future clinical treatment.

## Introduction

Urinary bladder cancer (BCa) is the fourth common malign neoplasia globally, responding for an incidence of approximately 7% among all male malignant tumours [1]. Pathologically, BCa can be manifested as low grade or high grade. In addition, according to the invasion of the muscular layer of the bladder wall, it also can be divided into non-muscular invasion or muscular invasion [2]. However, there is a large probability of false positives for the common screening method for BCa, such as urine cytology, due to its relatively low sensitivity [3]. Moreover, the diagnosis and post-treatment supervising of BCa requires expensive imaging and invasive cystoscopy, which patients need to undergo regularly several times each year to determine recurrence [4]. Therefore, it is urgently needed to investigate more convenient, non- or least invasion and accurate test for early diagnosis and predicting the prognosis for BCa patients [5].

As the standard and traditional method of initial treatment for patients with inoperable locally advanced or metastatic BCa, chemotherapy brings relatively high initial response rate while the median survival time is only about 15 months [5]. In addition, due to potential renal insufficiency, poor functional status or comorbidities, up to 50% of patients with advanced BCa could not receive cisplatin therapy. Since 2016, the emergence of new therapies such as immunotherapy and ADCs has broken this dilemma and brought significantly improved survival benefits for BCa patients [6]. With the progress of a series of clinical trials and data mining results, increasing evidence points to biomarkers, including PD-L1, TMB, dMMR/MSI-H, haemoglobin, etc., and predictive models predicting immune checkpoint therapies (ICTs) response [7–9]. However, there are more potential biomarkers for disease diagnosis and progression prediction worth exploring, and their application in the clinical strategies still needs to undergo multi-step verification [10].

To improve the accuracy of precision medicine, researchers have found several detectable and promising biomarkers of BCa [10,11]. However, due to instability of urine dilution, different races, environments and dietary culture, it is difficult for metabonomic in urine to become biomarkers for BCa [12]. Many tumour suppressors, such as Calcium activated chloride channel A4 (CLCA4), have been widely accepted as a marker related to tumour progression, including BCa [13]. Moreover, increasing evidence has indicated that high/low expression of some proteins or mutation of genes significantly correlated with carcinogenesis, progression and poor outcomes of BCa [14–16]. However, these biomarkers are not as accurate due to their genome instability or other inherent limitations [17]. Thus, novel biomarkers or prediction model that could be more accurate, convenient and sensitive for BCa patients is urgently needed.

Multi-omics, such as genomics, proteomics and single-cell omics techniques, are widely used for different purposes in different research fields, such as detecting disease-related diagnostic markers, understanding pathogenesis and explaining functional protein pathways in different diseases [18–20]. Recently, proteomic analysis has been accepted as a powerful tool to investigate the protein expression in different tissues or organs [21,22], and explore potential protein biomarkers for several cancers [23]. So, it is promising to investigate potential biomarkers and evaluate protein markers with economy and sensitivity in BCa using the reverse-phase protein arrays (RPPA) as a powerful approach of proteomic [24,25].

This study first identified landscape of novel prognostic protein signatures in the discovering dataset based on large-scale RPPA data (*n* = 340). Further, potential integrated prognosis-related proteins (IPRPs) model was constructed to assess the survival risk score of bladder cancer. Next, we tested the clinical relevance of baseline SRC protein and mRNA expression in two independent confirmatory cohorts (TCGA, *n* = 404; GSE13507, *n* = 162) and prognostic role of SRC in cancers. Then, we found that elevated SRC expression contributed to immunosuppressive microenvironment mediated by immune checkpoint molecules of BCa. Next, we validated SRC expression as a potential biomarker in predicting response to ICT in real-world cohort (*n* = 81). We found that the expression of SRC in the baseline tumour tissues correlated with improved survival benefits, but predicts worse ICT response. Cumulatively, this study revealed that the risk score of IPRPs model based on large-scale multi-omics data predicts outcomes for BCa patients. SRC, the key biomarker for better prognosis, may inversely improve predictive outcomes for patients receiving ICT and enable patient selection for ICT.

## Materials and methods

### Raw biological microarray data

The level 4 data of RPPA from the Cancer Proteome Atlas (TCPA) were recruited in analyses. Clinical data and gene expression profiles were obtained from the Cancer Genome Atlas (TCGA) for patients with BCa. A total of 341 BCa cases with full data of biological microarray data and clinical data were obtained using R software to impute the missing data and match the sample ID.

### Collected patients from a real-world cohort

The Fudan University Shanghai Cancer Centre (FUSCC, Guangxi, China) cohort consisted of 81 BCa patients receiving ICTs in the Department of Urology, Fudan University Shanghai Cancer Centre, from August 2018 to June 2020. Pathology reports or electronic medical records provided clinicopathological information. Samples of BCa and normal bladder tissues were collected during surgery and then processed and stored at the FUSCC tissue bank.

### Screening of candidate proteins

To assess the prognostic value of proteins, survival analysis was performed using the Kaplan–Meier method and the hazard ratio (HR) estimates with 95% confidence intervals (95% CI) were performed using univariate Cox regression analysis. The volcano plot and survival curves were plotted using ggplot2 package [26] and survival package [27] of R software, respectively. To elevate the prognostic accuracy, Lasso Cox regression analysis were performed to further restrict the candidate proteins using the glmnet package [28] of R language.

### Construction of multivariate cox model and IPRPs model

Multivariate analysis was utilized to identify the candidate proteins with most prognostic value and calculate the risk score based on the expression of proteins and survival rates. Risk level of patients was divided into high risk or low risk according to the median-risk score. To verify the correlation between patient’s prognosis and risk score, survival curves and scatter diagrams were plotted using R software. The heat map was drawn to visualize the expression of each candidate protein in two groups.

Univariate and multivariate Cox regression analyses were performed to identify the independent prognostic factors and construct the IPRPs model on this basis. The diagnostic stability of IPRPs model was assessed by receiver operating characteristic (ROC) and the corresponding area under curve (AUC). A cut-off of 0.4 for Pearson’s correlation was utilized to determine the proteins associated with IPRPs model. *p*-values for both statistics less than .05 were considered significant.

### Validation of proteomic prognostic value of hub nodes in genomic dataset

Gene expression data was utilized to further verify the prognostic value of proteomic results. A total of 404 BCa patients with available survival data were downloaded from TCGA and 165 BCa patients were enrolled in analysis from Gene Expression Omnibus (GEO) (Chip dataset GSE13507). Pearson correlation analysis were utilized to validate the correlations between protein and RNA expression. Survival analysis was performed using the Kaplan–Meier method and *p* value <.05 was considered significant. Additionally, the single-cell RNA-seq datasets, GSE145281 and GSE130001, were enrolled in this study from Tumour Immune Single-cell Hub to characterize tumour microenvironment at single-cell resolution [29].

### Gene set enrichment analysis and differential expressed genes (DEGs)

Gene Set Enrichment Analysis (GSEA) was utilized to investigate the potential hallmarks using transcriptional profiles from TCGA datasets between high- and low-risk group with 1000 permutations. Adjusted settings and parameters for GSEA and DEGs are as reported in previous studies [30–32]. Protein–protein interaction (PPI) networks of DEGs was predicted using Search Tool for the Retrieval of Interacting Genes (STRING; http://string-db.org) (version 10.0) [33]. An interaction with specificity scores high than 0.4 was regarded as statistically significant. To visualize molecular interaction networks of DEGs, Cytoscape (version 3.5) [34] and MCODE (version 1.4.2) [35], a Cytoscape plug-in, were utilized to select the most significant hub genes with MCODE Score ≥20. Functional enrichment analyses of hub genes were performed using ClusterProfiler package [36].

### Immunohistochemistry staining and evaluation

Immunohistochemistry (IHC) analysis was performed to detect implications of SRC expression in response to immunotherapy for BCa patients. SRC protein expression was detected in BCa tissues using anti-SRC (HPA030875) from the HPA database and Anti-SRC antibody (ab109381, Abcam, Cambridge, MA) from FUSCC cohort. Positive or negative staining of a certain protein in one FFPE slide was independently assessed by two experienced pathologists, and determined as previously described [37].

### Statistical analysis

All statistical analyses and graphical plotting were performed with SPSS (version 23.0, SPSS, Chicago, IL), GraphPad Prism 8 or R software (version 3.3.2, GraphPad Software, La Jolla, CA). Survival curves were established using the Kaplan–Meier method and analyzed by log-rank test with 95% confidence intervals (95% CI). All hypothetical tests were two-sided and *p* value less than .05 were considered statistically significant.

## Results

### Screening and identification of significantly prognostic proteins

**Figure 1.**
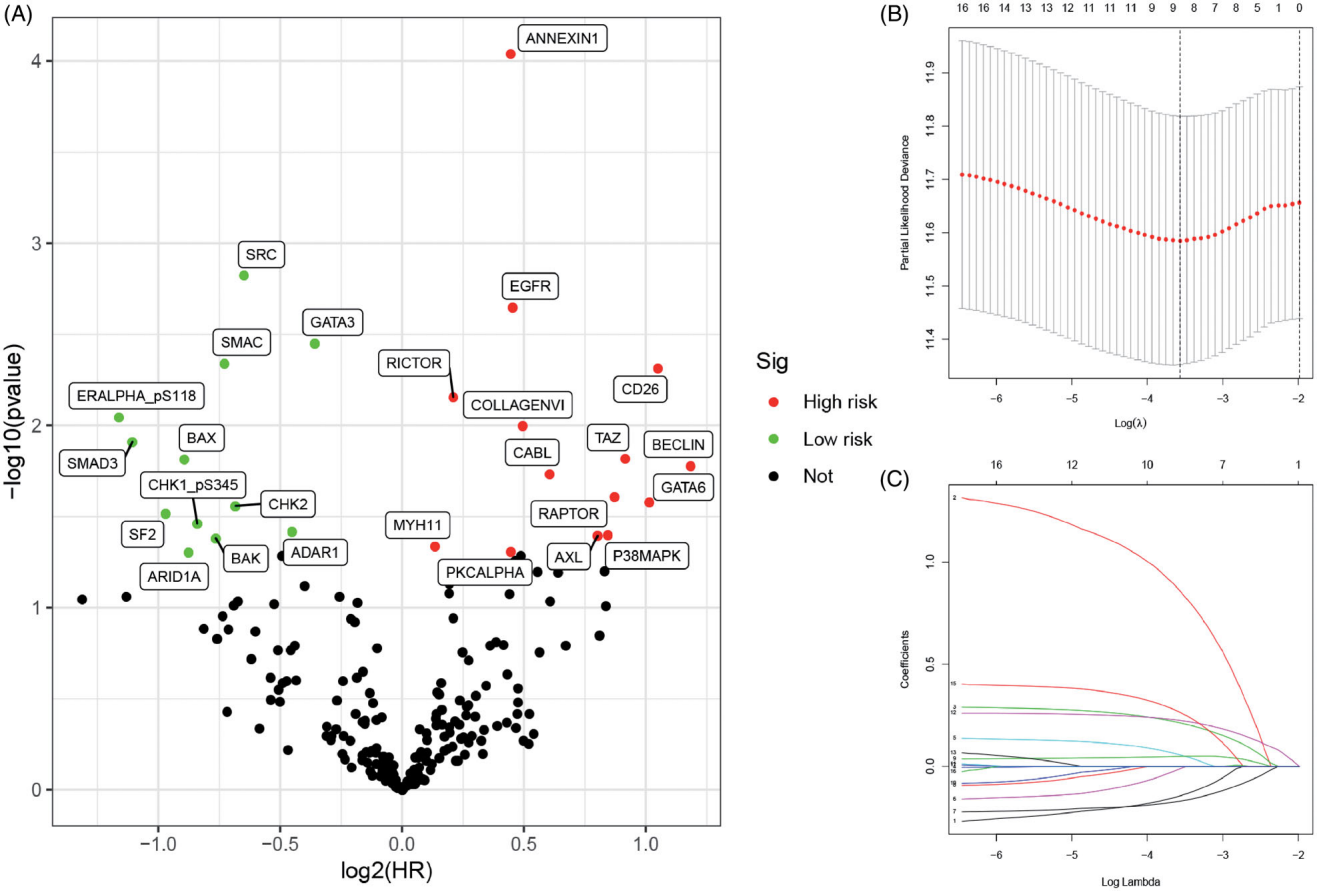
Selecting and identification of significantly prognostic proteins based on machine-learning algorithm in proteomics. (A) Volcano plot indicated the significantly prognostic proteins with high-risk (marked in red) and low-risk (marked in green). (B and C) Lasso Cox regression analysis was applied to further restrict the fit with prognostic values to obtain integrated prognosis-related proteins.

After identification and standardization of the microarray results, we performed Kaplan–Meier analysis and univariate Cox regression analysis to initially screen the 17 proteins as listed in Table 1. Selected proteins were separated as high- and low-risk, as shown in volcano plot (Figure 1(A)). LASSO Cox regression analysis further restricted the candidate proteins and nine proteins (BAK, BECLIN, EGFR, PKCALPHA, SMD3, SRC, RICTOR, ANNEXIN1 and AXL) were identified as prognostic proteins in BCa (Figure 1(B,C)). Ultimately, through multivariate Cox regression analysis, six proteins with the most prognostic value were identified and the risk score for each patient were calculated (integrated risk score= 1.285 × BECLIN expression (ref. Low) + 0.286 × EGFR expression (ref. low) + 0.242 × PKCALPHA expression (ref. low) − 0.240 × SRC expression (ref. low) + 0.293 × ANNEXIN1 expression (ref. low) + 0.556 × AXL expression (ref. low)).

**Table 1.** Kaplan–Meier analysis and univariate Cox regression analysis of differential expressed proteins (both *p*-value < 0.05).

Protein	*p*-value (KM)	*p*-value (Unicox)	HR
BAK	.001	.042	0.589 (0.354–0.979)
BECLIN	.027	.017	2.272 (1.160–4.448)
EGFR	.002	.002	1.369 (1.119–1.675)
GATA3	.001	.004	0.781 (0.662–0.922)
PKCALPHA	.044	.049	1.363 (1.001–1.855)
SMAD3	.036	.012	0.464 (0.255–0.847)
SRC	.001	.002	0.639 (0.484–0.842)
ARID1A	.002	.050	0.545 (0.297–1.000)
RICTOR	.017	.007	1.157 (1.041–1.287)
SF2	<.001	.030	0.510 (0.278–0.938)
TAZ	<.001	.015	1.887 (1.130–3.151)
ANNEXIN1	<.001	<.001	1.362 (1.167–1.590)
ADAR1	.035	.038	0.731 (0.544–0.983)
SMAC	.014	.005	0.604 (0.426–0.856)
AXL	.002	.040	1.744 (1.025–2.969)
GATA6	.003	.026	2.020 (1.086–3.759)
CABL	.004	.018	1.522 (1.073–2.160)

KM: Kaplan–Meier analysis; Unicox: univariate Cox regression analysis; HR: hazard ratio.

### Survival analysis and construction of IPRPs model

**Figure 2.**
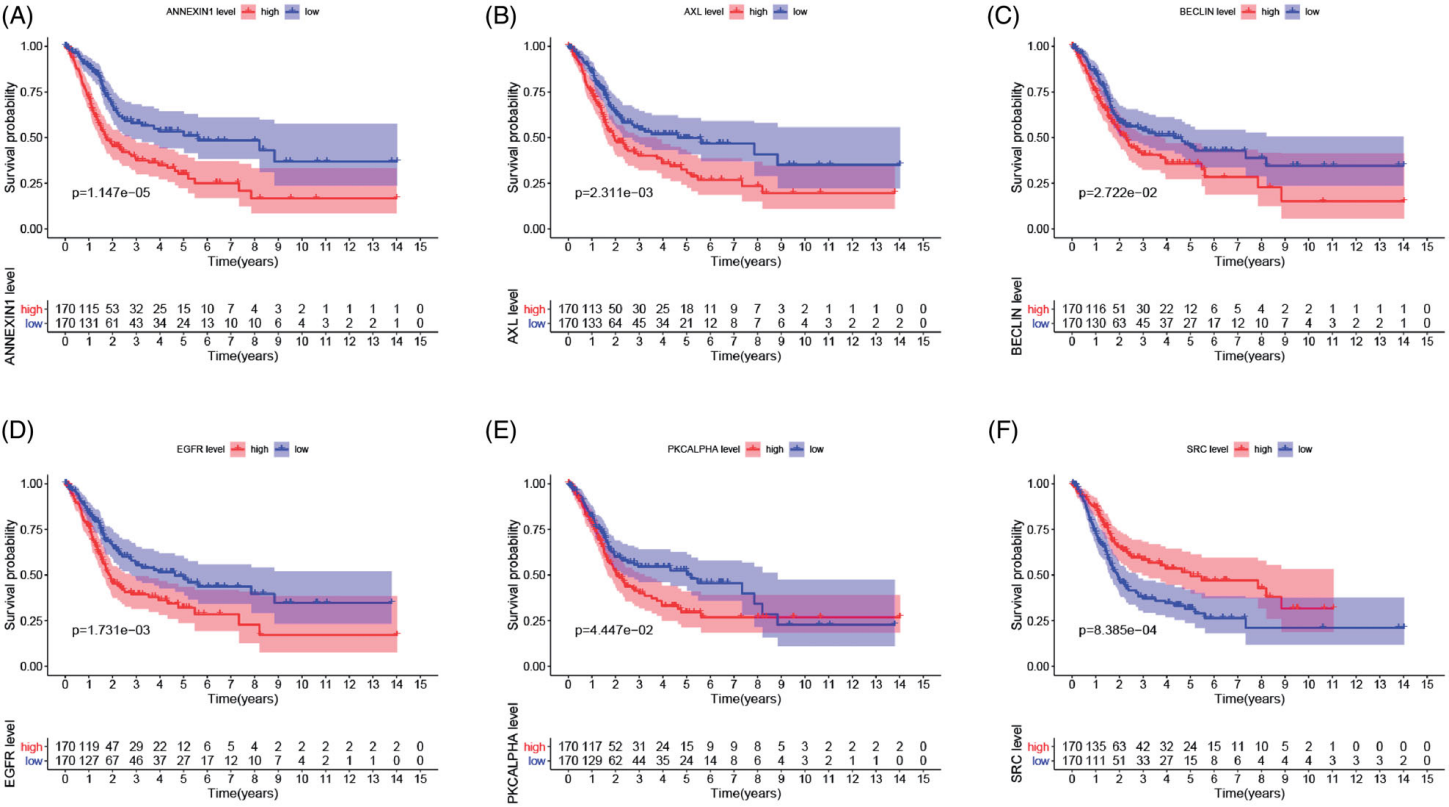
Survival analysis indicated significantly prognostic role of BECLIN, EGFR, PKCALPHA, ANNEXIN1, AXL, SRC proteins expression. (A–E) High expression of BECLIN, EGFR, PKCALPHA, ANNEXIN1 and AXL significantly correlated with worse outcomes in BCa patients (*p* < .05). (F) Down-regulated of SRC was markedly related with poor prognosis (*p* < .001).

**Figure 3.**
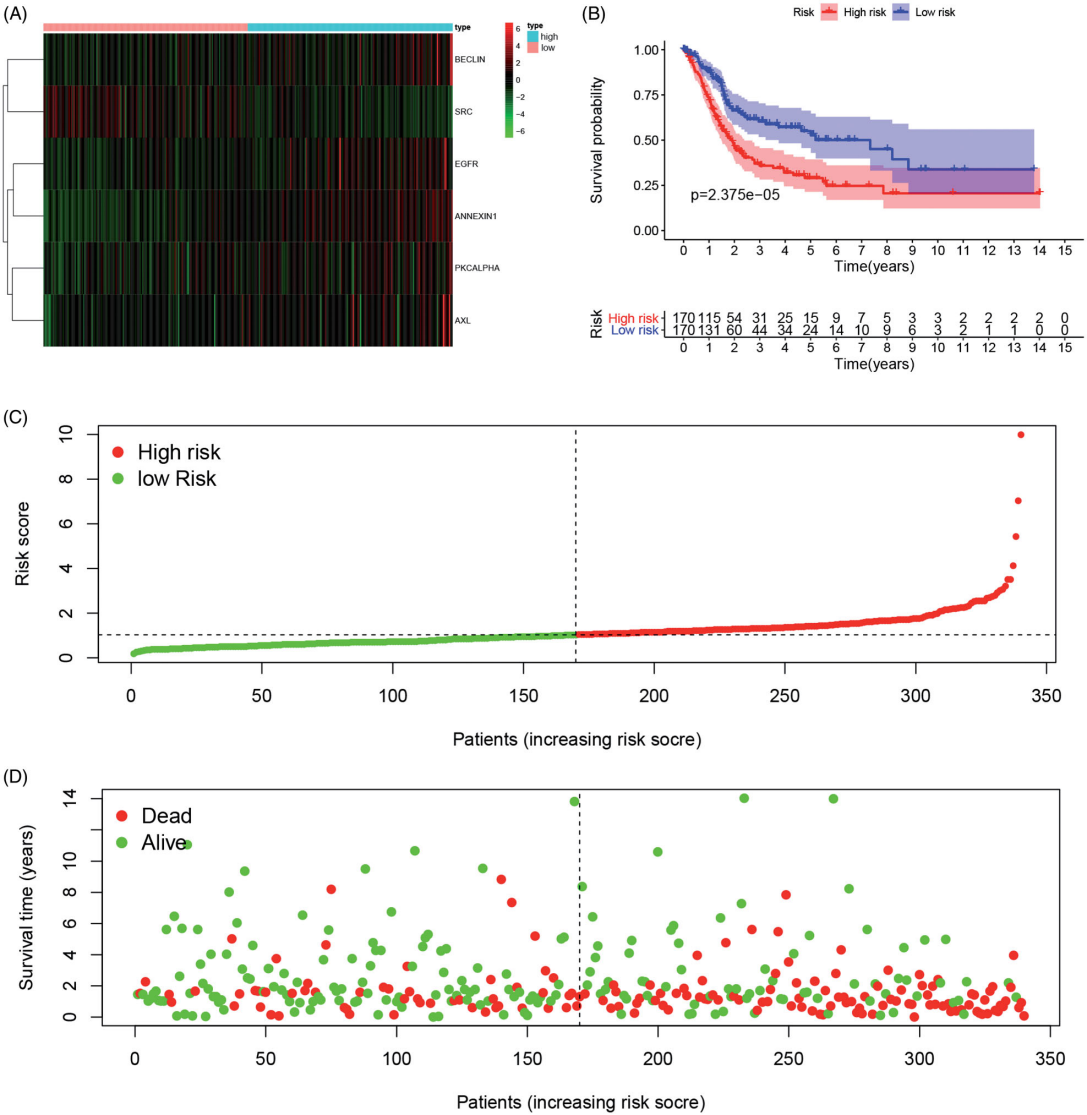
Differential expression of integrated prognosis-related proteins (IPRPs) and construction of the prediction model. (A) Expression of each IPRPs in high- and low-risk BCa groups. (B) Survival analysis showed strong power of IPRPs model to predict prognosis that high-risk BCa patients had significantly worse prognosis comparing to low-risk group (*p* < 0.001). (C and D) Increased risk score responded to shorter survival cases.

As shown in Figure 2, survival curves demonstrated that elevated BECLIN, EGFR, PKCALPHA, ANNEXIN1, AXL expression and down-regulation of SRC significantly correlated with poor outcomes of BCa patients (*p* < .05), and identified as IPRPs. In addition, the expression of six proteins between the high- and the low-risk group displayed in the heatmap (Figure 3(A)). Survival analysis showed strong power of IPRPs model to predict prognosis that high-risk BCa patients had significantly worse prognosis comparing to the low-risk group (*p* < .001; Figure 3(B)). Patients in the high-risk group had poor survival outcome comparing to the low-risk group (*p* < .01) and the increased risk score responded to shorter survival cases (Figure 3(C,D)).

### Independent prognostic implication of IPRPs model

**Figure 4.**
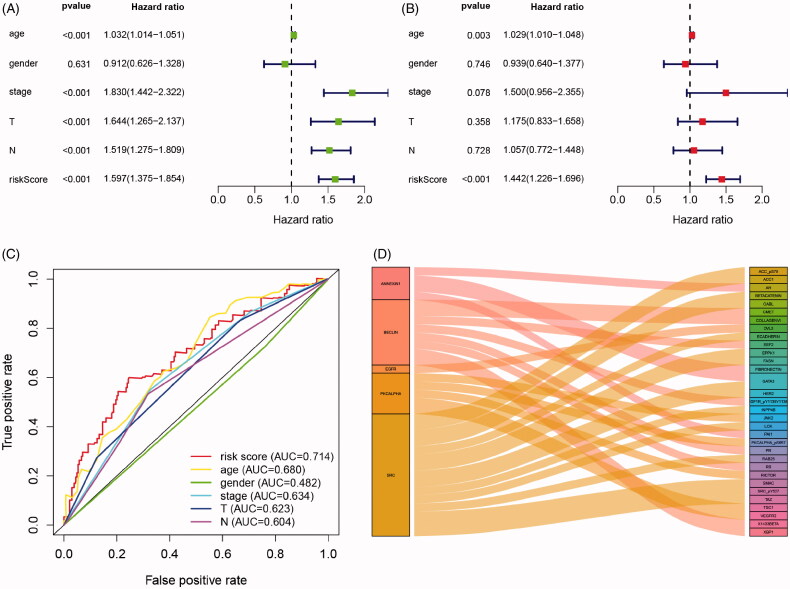
Cox regression and ROC analysis indicated significantly and independently prognostic role of IPRPs model. (A and B) Forest plot displayed that the risk score of IPRPs model significantly predicts survival for BCa patients in univariate (*p* < .001, HR = 1.597) and multivariate (*p* < .001, HR = 1.442) Cox regression analysis, respectively. (C) ROC curves showed the predictive ability of each independent factor, and the risk score exhibited a good prognostic value with AUC = 0.714. (D) Sankey diagram showed the association between various types of proteins and IPRPs.

Cox regression analysis showed that in univariate models, traditional prognostic factors, specifically pT stage, pN stage and pathologic stage were significantly correlated with OS (all *p* < .001). Importantly, in both univariate and multivariate models, risk score of IPRPs model was significantly relevant to OS (univariate: HR = 1.597, *p* < .001; multivariate: HR = 1.443, *p* < .001; Figure 4(A,B)). In addition, the age of patients was also significantly correlated with OS both in univariate (*p* < .001) and multivariate (*p* = .003) models. Moreover, As shown in Figure 4(C), age, T stage and N stage had a potential prediction value with all AUC > 0.500; notably, the risk score of IPRPs model was particular exhibited high diagnostic accuracy and consistent predictive ability (AUC = 0.714).

### Significant proteins associated with IPRPs

Sankey diagram showed the association between various types of proteins and IPRPs (Figure 4(D)). A total of 8 proteins significantly correlated with the expression of BECLIN (correlation coefficients range from −0.43 to 0.59, *p* < .001), and 5 with the expression of PKCALPHA (correlation coefficients range from 0.41 to 0.94, *p* < .001), 4 with ANNEXIN1 (correlation coefficients range from −0.447 to 0.42, *p* < .001), 15 with SRC (correlation coefficients range from −0.48 to 0.61, *p* < .001), only 1 with EGFR (correlation coefficient = 0.48, *p* < .001), and the detailed displayed in Supplementary Figure 1.

### Genomic prognostic validation of significant proteins

**Figure 5.**
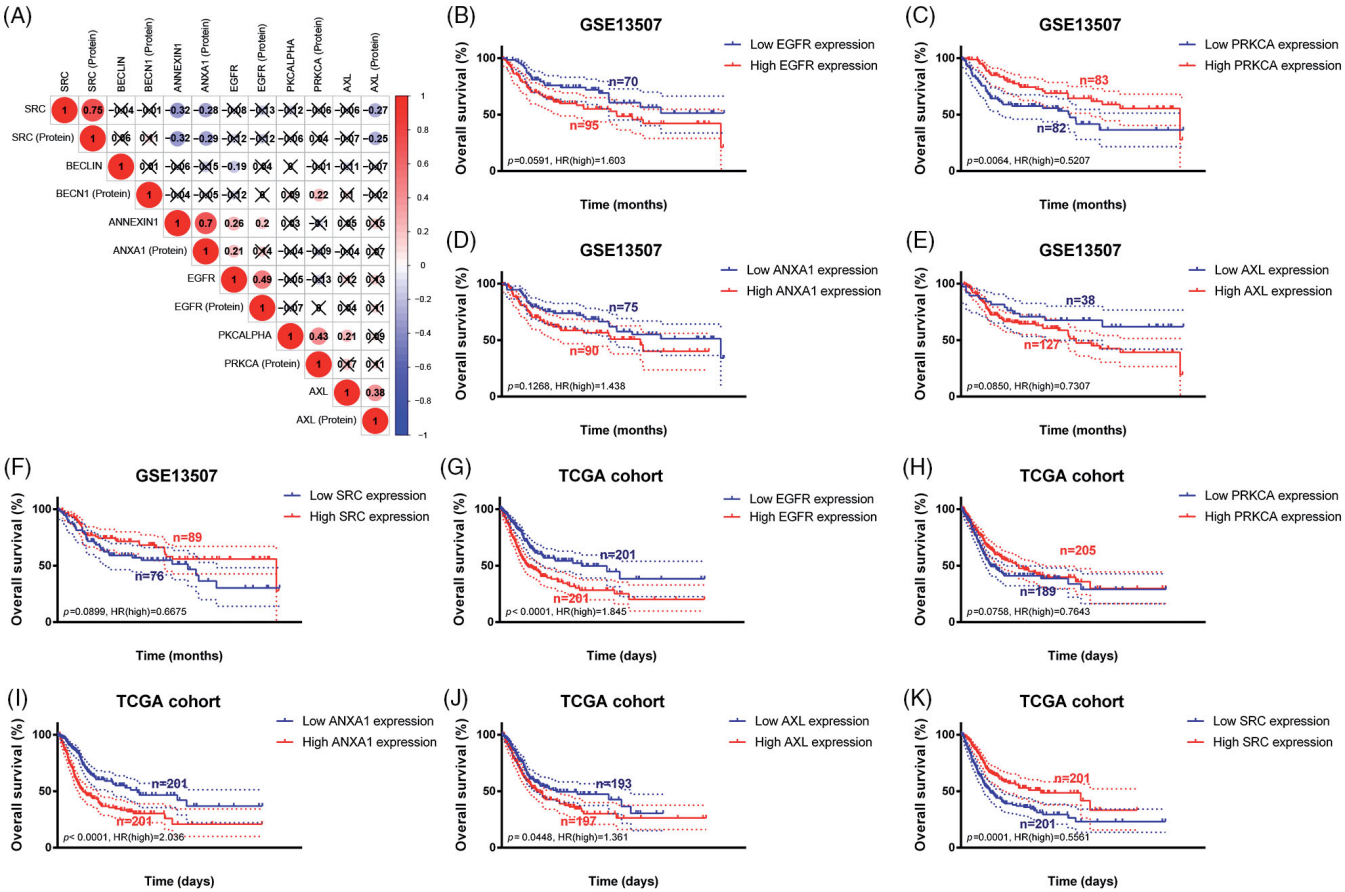
Genomic prognostic validation of significant proteins in multiply datasets. (A) Proteome expression of EGFR, PKCALPHA, ANNEXIN1, AXL and SRC were positively correlated with corresponding mRNA expression. (B–F) Survival analyses were performed in GEO and TCGA cohorts respectively, and the results indicated that low PRKCA (protein named PKCALPHA) expression was significantly correlated with OS in GSE13507 cohort (*p* = .006). (G-K) In larger sample TCGA cohort, high expression of EGFR, ANXA1 (protein named ANNEXIN1), AXL and low expression of SRC were markedly related with poor outcomes, which is consistent with results of proteomics (*p* < .05).

To further validate the prognostic value of six significant proteins, we first performed Pearson correlation analysis between RNA expression and protein expression. As shown in Figure 5(A), proteome expressions of EGFR, PKCALPHA, ANNEXIN1, AXL and SRC were positively correlated with corresponding mRNA expression. Second, survival analyses were performed in GEO and TCGA cohorts, respectively, and the results indicated that low PRKCA (protein name is PKCALPHA) expression was significantly correlated with OS in GSE13507 cohort (*p* = .006) (Figure 5(B–F)). More importantly, in larger sample TCGA cohort, high expression of EGFR, ANXA1 (protein name is ANNEXIN1), AXL and low expression of SRC were markedly related with poor outcomes, which is consistent with results of proteomics (all *p* < .05) (Figure 5(G–K)).

### Significant involved genes and pathways related to IPRPs model

**Figure 6.**
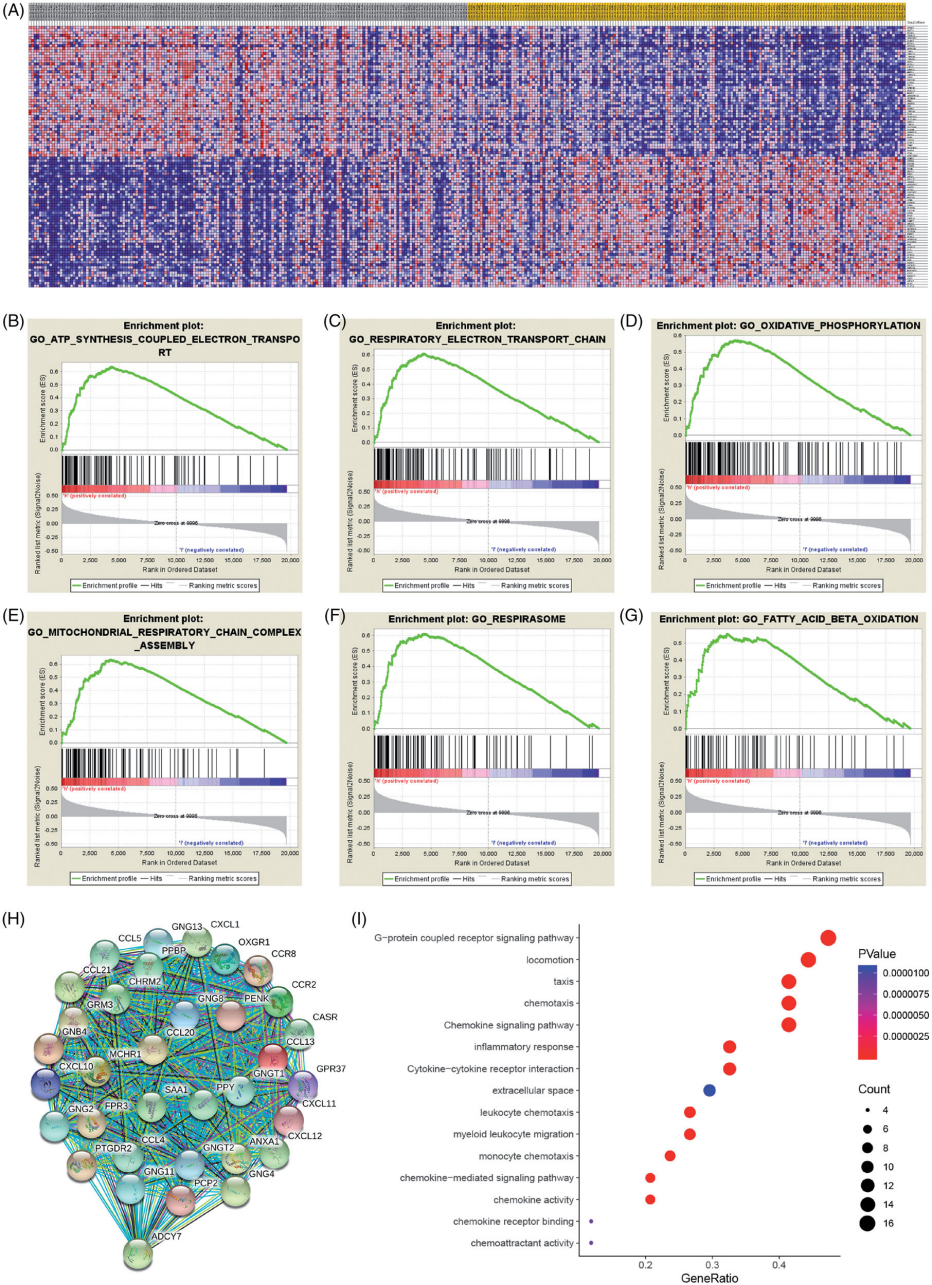
Significant involved genes and hallmarks pathways of IPRPs model in BCa obtained from GSEA functional enrichment analysis. (A) Top 100 significant genes positively and negatively correlated with risk score of IPRPs model were performed in a heat map. (B–G) The most involved significant pathways included ATP synthesis coupled electron transport, respiratory electron transport chain, oxidative phosphorylation, mitochondrial respiratory chain complex assembly, respirasome and fatty acid beta oxidation. (H) The protein-protein interaction network of differential expressed genes was constructed and visualize. (I) Functional enrichment analyses of hub genes were performed and visualized in bubble chart. Significant genes were enriched in G-protein coupled receptor signalling pathway, locomotion, taxis and chemokine signalling pathways (*p* < .0001).

**Figure 7.**
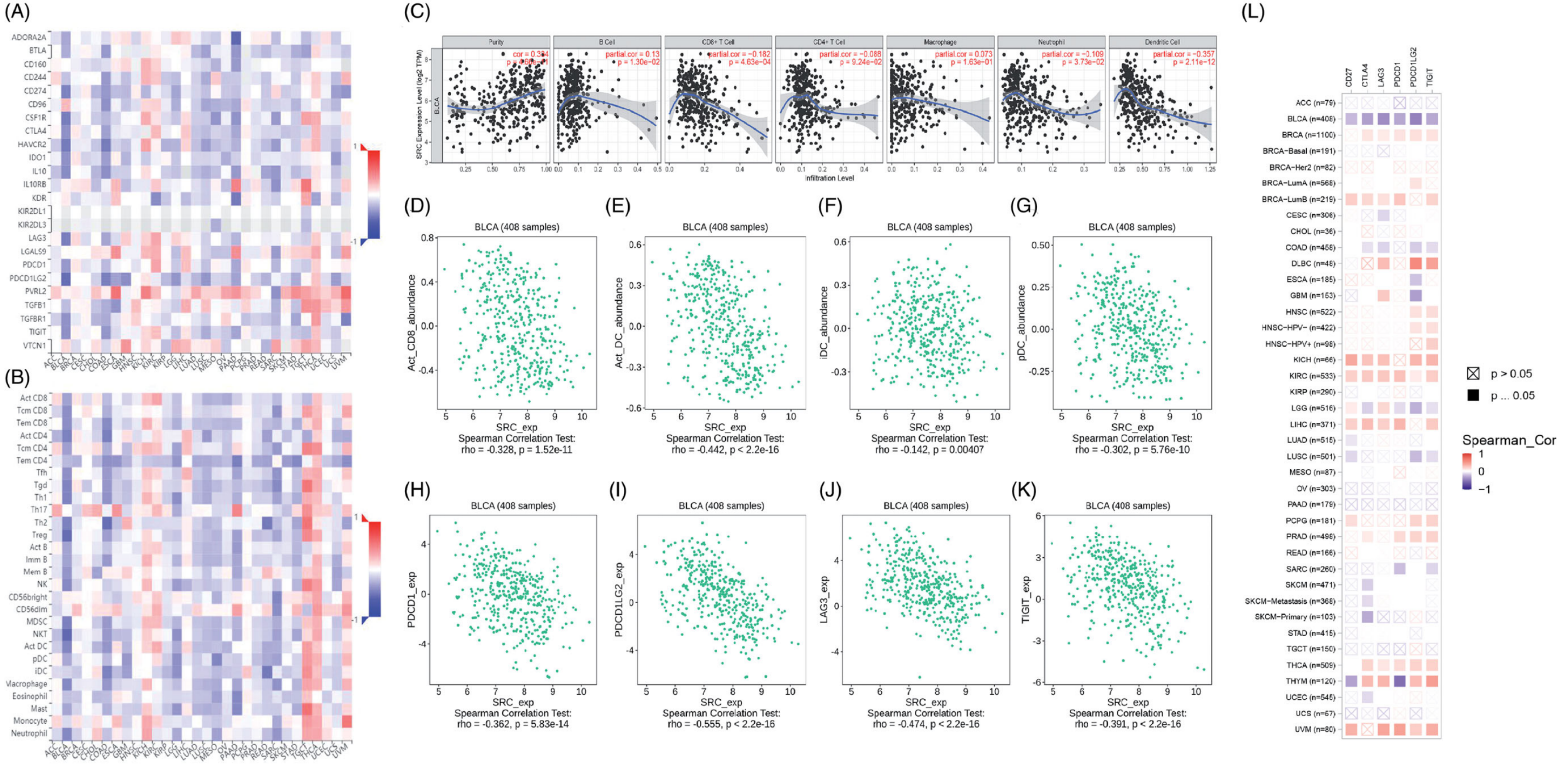
SRC correlate with immune cell infiltrations pan-cancers microenvironment and negatively correlated with expression levels of immune check-point molecular in BCa. (A-B) Spearman correlations between SRC and immunoinhibitors and abundance of tumour-infiltrating lymphocytes (TILs) across human cancers were applied. SRC expression were negatively correlated with immunoinhibitors expression and abundance of TILs in BCa. (C) SRC expression showed significantly positive associated with tumour purity (cor.=0.334), and negative association with CD8^+^ T cell (cor.= −0.182) and dendritic cell infiltration (cor.= −0.357). (D–G) Spearman’ test suggested that SRC expression significantly correlated with decreased abundance of activated CD8^+^ T cells (*r*= −0.328), activated dendritic cells (*r*= −0.442), immature dendritic cells (*r*= −0.142) and plasmacytoid dendritic cells (*r*= −0.302). (H–K) Spearman’ test suggested that SRC expression significantly correlated PDCD1 (*r*= −0.362), PDCD1LG2 (*r*= −0.555), LAG3 (*r*= −0.474) and TIGIT (*r*= −0.391). (L) Spearman’s correlation tests indicated that SRC significantly showed negative correlation with immune check-point molecular expressions, which differs from other cancers.

As shown in heatmap (Figure 6(A)), a total of most 100 significant genes obtained from GSEA with positive and negative correlations. GSEA demonstrated that significant pathways of IPRPs model mainly involved in ATP synthesis coupled electron transport, respiratory electron transport chain, oxidative phosphorylation, mitochondrial respiratory chain complex assembly, respirasome and fatty acid beta oxidation (Figure 6(B–G)). Additionally, a total of 917 DEGs associated with risk score were identified and protein–protein interaction network of DEGs was constructed and selected 29 hub genes (PPY, CXCL11, CASR, CXCL10, ADCY7, CCL5, MCHR1, OXGR1, CCL20, CXCL1, CCL21, PCP2, CCR8, CXCL12, CCR2, GRM3, CHRM2, CCL13, GPR37, FPR3, ANXA1, SAA1, CCL4, GNG8, GNB4, GNG4, PTGDR2, PENK, PPBP). The protein–protein interaction network of differential expressed genes was constructed and visualize in Figure 7(H). Functional enrichment analyses of hub genes were performed and visualized in bubble chart. Significant genes were enriched in G-protein coupled receptor signalling pathway, locomotion, taxis and chemokine signalling pathways (*p* < .0001; Table 2; Figure 6(I)).

**Table 2.** GO and KEGG pathways enrichment analysis of hub genes.

Term	Description	Count in gene set	*p* value
GO:0006935	Chemotaxis	14	1.77E-14
GO:0042330	Taxis	14	1.77E-14
GO:0007186	G-protein coupled receptor signalling pathway	16	2.13E-13
GO:0002548	Monocyte chemotaxis	8	7.16E-13
GO:0097529	Myeloid leukocyte migration	9	2.00E-11
GO:0030595	Leukocyte chemotaxis	9	8.97E-11
GO:0070098	Chemokine-mediated signalling pathway	7	1.59E-10
GO:0006954	Inflammatory response	11	1.97E-10
GO:0040011	Locomotion	15	5.59E-10
GO:0005615	Extracellular space	10	1.06E-05
GO:0008009	Chemokine activity	7	1.71E-11
GO:0048020	Chemokine receptor binding	4	8.81E-06
GO:0042056	Chemoattractant activity	4	8.81E-06
hsa04062	Chemokine signalling pathway	14	2.07E-18
hsa04060	Cytokine–cytokine receptor interaction	11	5.53E-12

GO: Gene Ontology; KEGG: Kyoto Encyclopaedia of Genes and Genomes.

### SRC correlate with immune cell infiltrations in BCa and pan-cancers

Spearman correlations between SRC and immunoinhibitors and abundance of tumour-infiltrating lymphocytes (TILs) across human cancers were applied. SRC expressions were negatively correlated with immunoinhibitors expression and abundance of TILs in BCa (Figure 7(A,B)). As shown in Figure 7(C), SRC expression showed significantly positive associated with tumour purity (cor.=0.334), and negative association with CD8+ T cell (cor.= −0.182) and dendritic cell infiltration (cor.= −0.357). In Figure 7(D–G), Spearman’ test suggested that SRC expression significantly correlated with decreased abundance of activated CD8+ T cells (*r*= −0.328), activated dendritic cells (*r*= −0.442), immature dendritic cells (*r*= −0.142) and plasmacytoid dendritic cells (*r*= −0.302). In Figure 7(H–K), Spearman’ test suggested that SRC expression significantly correlated PDCD1 (*r*= −0.362), PDCD1LG2 (*r*= −0.555), LAG3 (*r*= −0.474) and TIGIT (*r*= −0.391). Next, we found SRC markedly correlated with immune checkpoint molecules in most cancers in TCGA database in Table S1. As shown in Figure 7(L), Spearman’s correlation tests indicated that SRC significantly showed negative correlation with immune check-point molecular expressions, which differs from other cancers.

### Prognostic implications of SRC expression in cancers in its subgroup survival analysis of BCa

**Figure 8.**
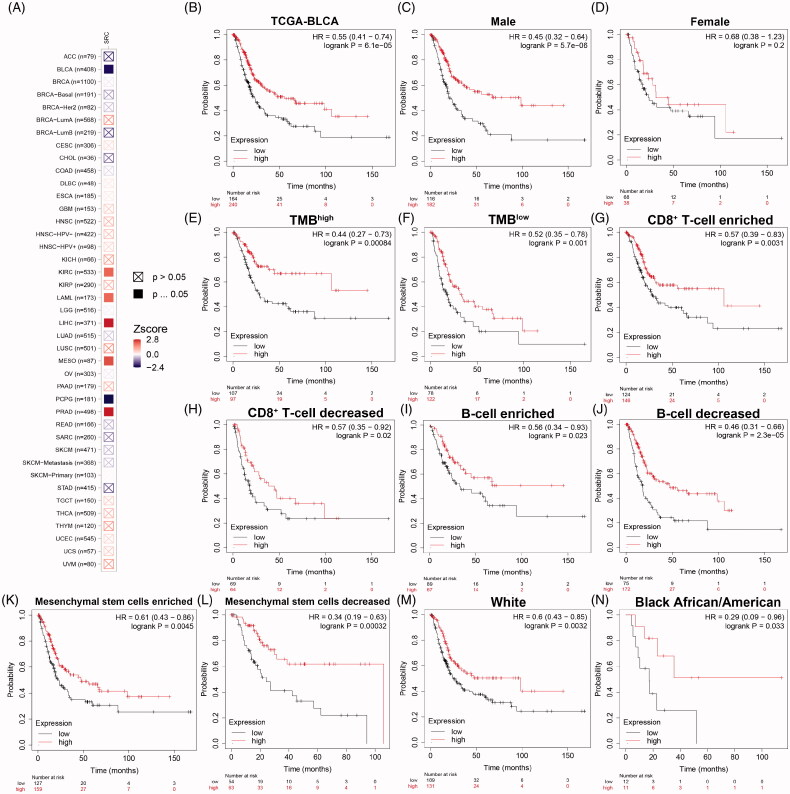
Prognostic implications of SRC expression in cancers in its subgroup survival analysis of BCa. (A) Prognostic role of SRC in cancers, with significance marked in solid. (B) Survival analysis showed significantly prognostic role of SRC in TCGA-BLCA cohort (*p* < .001, HR = 0.55). (C and D) Decreased SRC expression significantly correlated poorer outcomes in male (*p* < 0.001, HR = 0.45), while have no significant association with prognosis in female BCa patients (*p* = 0.2). (E and F) Decreased SRC expression significantly correlated poorer outcomes in both high (HR = 0.44) and low (HR = 0.52) tumour mutation burden. (G–J) Decreased SRC expression significantly correlated poorer outcomes in CD8^+^ T cells infiltration enriched (HR = 0.57) and decreased (HR = 0.57) subgroups, and B cells infiltration enriched (HR = 0.56) and decreased (HR = 0.46) subgroups. (K and L) Decreased SRC expression significantly correlated poorer outcomes in mesenchymal stem cells enriched (HR = 0.61) and, especially, decreased (HR = 0.34) subgroups. (M and N) Decreased SRC expression significantly correlated poorer outcomes in while (HR = 0.60) and black African or American (HR = 0.29) subgroups.

As shown in Figure 8(A), prognostic role of SRC was detected in cancers, with significance marked in solid. Prognostic value with *Z*-scores and adjusted *p*-value of SRC in cancers based on TCGA database using K–M and log-rank methods are shown in Table S2. Next, survival analysis showed significantly prognostic role of SRC in TCGA-BLCA cohort (*p* < 0.001, HR = 0.55; Figure 8(B)). In Figure 8(B,C), decreased SRC expression significantly correlated poorer outcomes in male (*p* < .001, HR = 0.45), while have no significant association with prognosis in female BCa patients (*p* = .2). In Figure 8(E,F), decreased SRC expression significantly correlated poorer outcomes in both high (HR = 0.44) and low (HR = 0.52) tumour mutation burden. In Figure 8(G–J), decreased SRC expression significantly correlated poorer outcomes in CD8^+^ T cells infiltration enriched (HR = 0.57) and decreased (HR = 0.57) subgroups, and B cells infiltration enriched (HR = 0.56) and decreased (HR = 0.46) subgroups. In Figure 8(K,L), decreased SRC expression significantly correlated poorer outcomes in mesenchymal stem cells enriched (HR = 0.61) and, especially, decreased (HR = 0.34) subgroups. In Figure 8(M,N), decreased SRC expression significantly correlated poorer outcomes in while (HR = 0.60) and black African or American (HR = 0.29) subgroups.

### SRC predicts predictive outcomes for BCa patients receiving ICT

**Figure 9.**
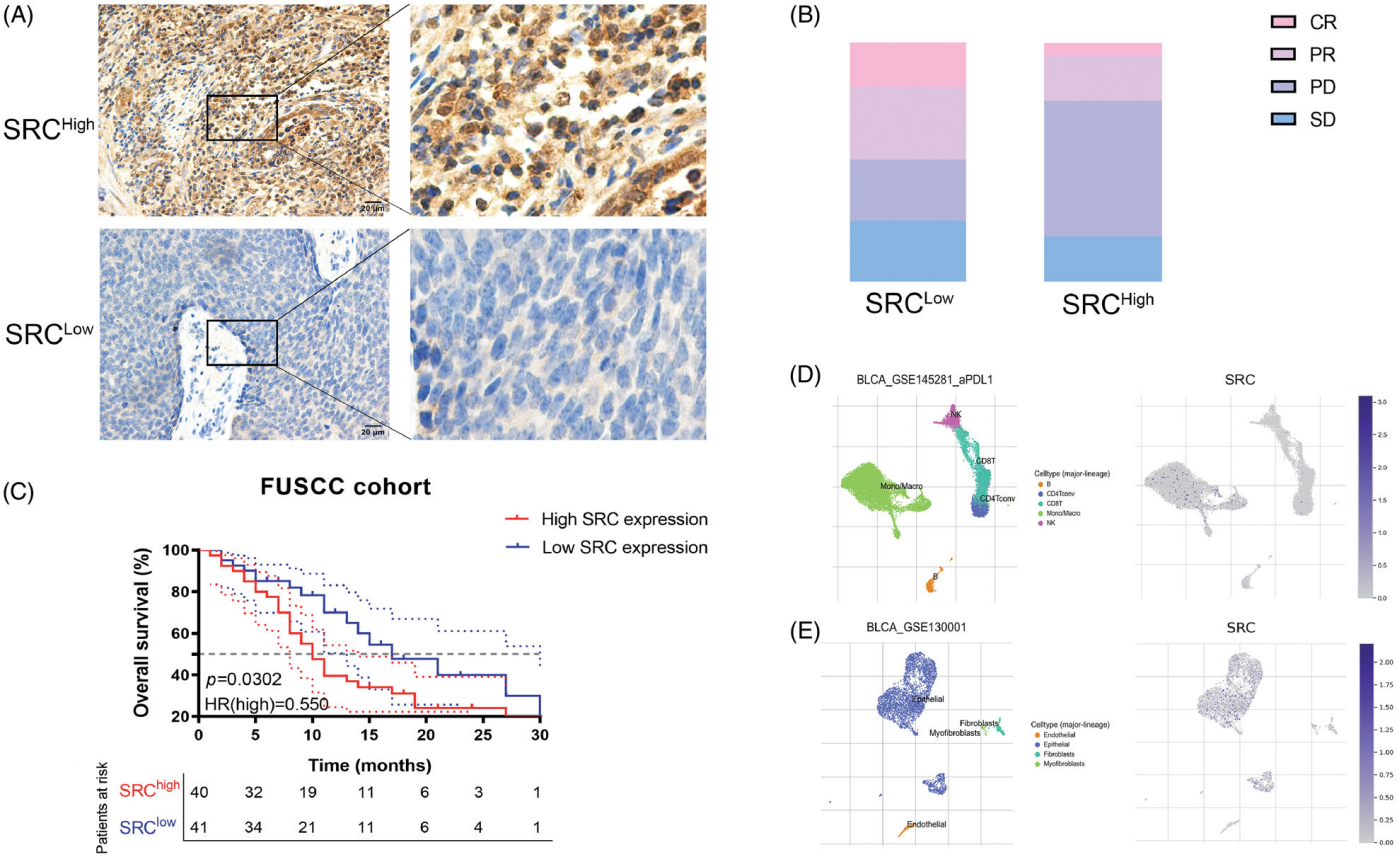
SRC protein expression significantly predicts immunotherapy responses for 81 BCa patients from FUSCC cohort. (A) Immunohistochemistry was performed and SRC was mainly stained in plasma membrane and cytosol. (B) Elevated SRC protein expression significantly correlated with poorer survival in 81 BCa patients receiving ICT. (C) There are 32% PR (*n* = 14), 18% CR (*n* = 8), 25% SD (*n* = 11) and 25% PD (*n* = 11) cases in low SRC expression subgroup, and 19% PR (*n* = 7), 5% CR (*n* = 2), 19% SD (*n* = 7) and 57% PD (*n* = 21) cases in high SRC expression subgroup. (D and E) The single-sequencing dataset GSE145281 and 130001 suggested localization and binding targets of SRC mainly in monocyte/macrophages and epithelial cells.

To detect implications of SRC expression in response to immunotherapy for BCa patients, immunohistochemistry (IHC) was performed. SRC protein expression was detected in BCa tissues using anti-SRC (HPA030875) from the HPA database and Anti-SRC antibody (ab109381) from FUSCC cohort. SRC was mainly stained in plasma membrane and cytosol (Figure 9(A); Supplementary Figure 2). Inversely, elevated SRC protein expression significantly correlated with poorer survival in 81 BCa patients receiving ICT from Fudan University Shanghai Cancer Centre (Figure 9(B)). There are 32% PR (*n* = 14), 18% CR (*n* = 8), 25% SD (*n* = 11) and 25% PD (*n* = 11) cases in the low SRC expression subgroup, and 19% PR (*n* = 7), 5% CR (*n* = 2), 19% SD (*n* = 7) and 57% PD (*n* = 21) cases in the high SRC expression subgroup (Figure 9(C)). Interestingly, the single-sequencing dataset GSE145281 and GSE130001 suggested localization and binding targets of SRC mainly in monocyte/macrophages and epithelial cells (Figure 9(D,E)).

## Discussion

Bladder cancer ranks 7th and 17th among the most common tumours in men and women, respectively, and is also an important public health problem in China [38]. There were 80,500 new cases of BCa, of which 32,900 cases responding for cancer-related death in 2015 [39]. In America, 19.5% of every 100,000 people suffer from bladder cancer, with a mortality rate of 7.9% [40], and US $5 billion is predicted to spend on the treatment of bladder cancer by 2020 [41]. With rapid development of precision medicine, there are more potential biomarkers for disease diagnosis and progression prediction worth exploring and applying in clinical strategies. Additionally, identification of baseline expression of a single gene evaluated by currently available matching learning methods would have immediate and promising translational potential.

This study first identified landscape of novel prognostic protein signatures in the discovering dataset based on large-scale RPPA data (*n* = 340). Next, potential integrated prognosis-related proteins (IPRPs) model (including BECLIN, PKCALPHA, EGFR, ANNEXIN1, AXL and SRC) was constructed to assess the survival risk score of bladder cancer. BECLIN is identified as a necessary autophagy protein, which has been proved to play an essential role in BECLIN ± mice tumour inhibition [42]. PKCALPHA involved in mediating cell proliferation, differentiation and apoptosis. In the process of cell function regulation, it interacts with many proto-oncogenes [43]. The expression and activation of EGFR are related to many precancerous lesions and malignant tumours [44], and is associated with malignancy of at least 33–50% of human epithelial tumours [45]. In addition, EGFR has been considered to be the carcinogenic driver of NSCLC and predicts TKI inhibitors therapy responses [46]. ANNEXIN1 is a member of the ANNEXIN protein superfamily, which not only has a very clear anti-inflammatory effect, but also plays an important role in apoptosis [47]. Importantly, ANNEXIN1 has been proved to be a key regulator of adaptive immunity through its ability to control T cell activation and autoimmune diseases [48]. Next, the overexpression of AXL is found closely related to cell proliferation, migration and invasion by activating carcinogenic signalling pathways, including PI3K/Akt and/or MAPK/Erk [49].

SRC is a non-receptor tyrosine kinase, which is activated following engagement of many different classes of cellular receptors and plays an important role in many kinds of carcinogenesis [50]. In addition, SRC inhibitors have been shown in preclinical trials that they may be used in targeted therapy for advanced breast cancer by inhibiting bone metastasis [51]. Our study also demonstrated that SRC, as the key biomarker for better prognosis, may inversely It is increasingly proven that the immune response to ICT is affected by immune cells and tumour-related factors. Activation of SRC family kinases may lead to elevated abundance of myeloid-derived suppressor cell, lack of anti-tumour immune responses and anti-CTLA4 immunotherapy resistance [52,53]. The combinatorial treatment with SRC family kinases inhibitors could increase the of anti-CTLA4 immunotherapy efficacy of HNSCC [52]. Interestingly, although elevated SRC expression predict favourable clinical outcomes, c-SRC kinase together with a therapeutic T-cell receptor blocked pMHC-induced ERK phosphorylation and turning T cells incapable of executing their downstream effector functions into "dummy T cell" [54]. These evidences fully indicated that SRC had great potential in predicting the efficacy for patients receiving ICTs.

However, this study also has its limitation. Our study is based on the retrospective design of large-scale proteomic and genomic analysis and failed to clarify the underlying mechanism of IPRPs. Multicenter prospective studies are needed to validate the results and prediction value of IPRPs model. In addition, it is still necessary to perform *in vitro* and *in vivo* experiments investigating role of SRC as potential therapeutic target and predictive biomarker for overall survival and ICT responses.

## Conclusion

This study first performed the large-scale multi-omics analysis, distinguished the IPRPs (*BECLIN, EGFR, PKCALPHA, SRC, ANNEXIN1* and *AXL*) and revealed novel prediction model, outperforming the currently traditional prognostic indicators for anticipating BCa progression and better clinical strategies. Additionally, this study provided insight into the importance of biomarker SRC for better prognosis, which may inversely improve predictive outcomes for patients receiving ICT and enable patient selection for future clinical treatment.

## Supplementary Material

Supplemental Material

## Data Availability

The datasets used and/or analyzed during the current study are available from the corresponding author on reasonable request or online database.
