# Supplementary figures and images for: Multi-omics reveals novel prognostic implication of SRC protein expression in bladder cancer and its correlation with immunotherapy response
Source: Ann Med. 2021 Apr 8;53(1):596–610. doi: 10.1080/07853890.2021.1908588 (PMC8043611; doi:10.1080/07853890.2021.1908588)

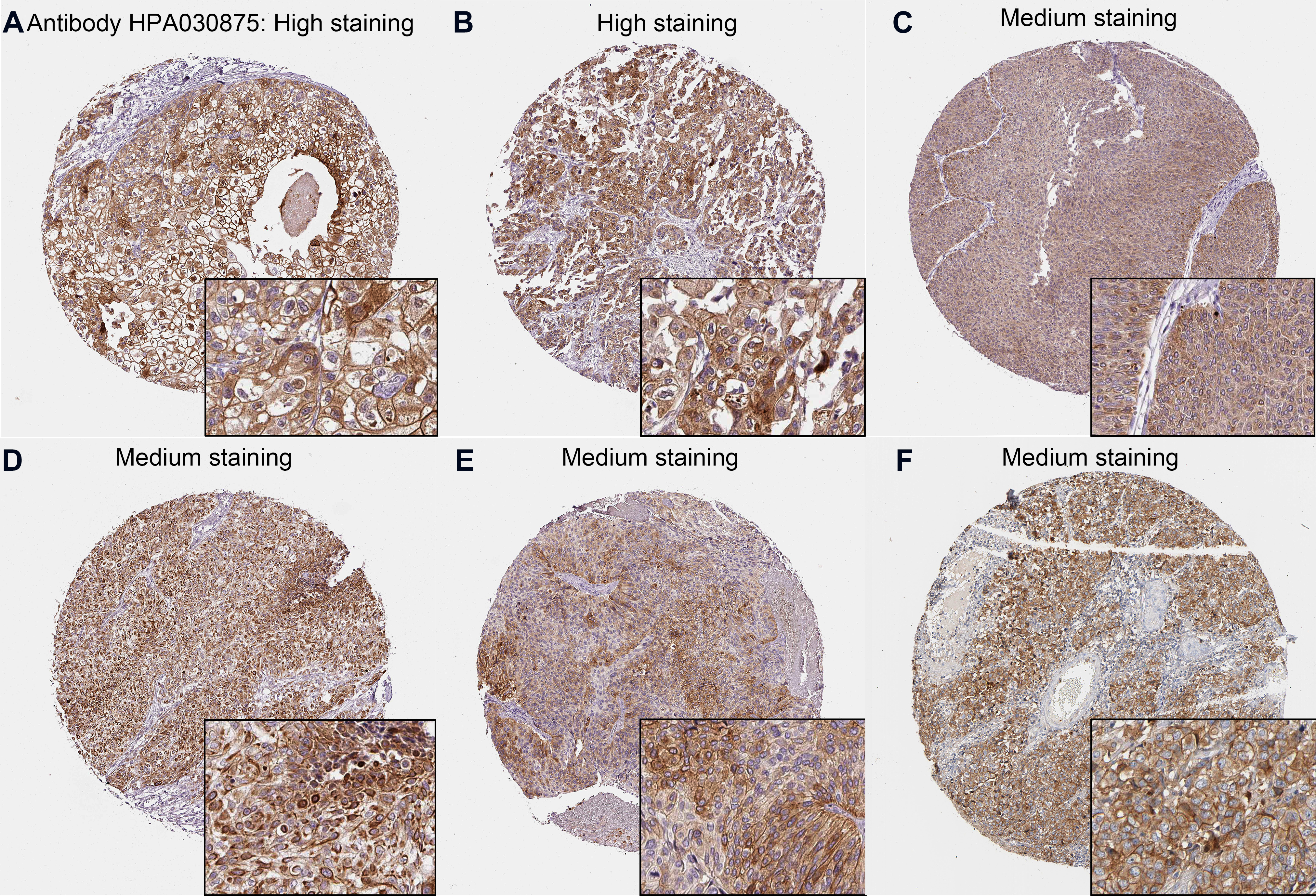

Supplement: Supplemental Material [file IANN_A_1908588_SM6846.zip › Supplemental files/Figure S2.tif]
